# Resolving the biological paradox of aneurysm formation in children with tuberous sclerosis complex

**DOI:** 10.1016/j.jvssci.2021.05.003

**Published:** 2021-05-24

**Authors:** Ulf Hedin, Hans Brunnström, Maria Dahlin, Torbjörn Backman, Valeria Perez de Sa, Phan-Kiet Tran

**Affiliations:** a Department of Vascular Surgery, Karolinska University Hospital, and Karolinska Institute, Stockholm, Sweden; b Molecular Medicine and Surgery, Karolinska University Hospital, and Karolinska Institute, Stockholm, Sweden; c Department of Clinical Sciences, Division of Pathology, Lund University and Skåne University Hospital, Lund, Sweden; d Department of Neuropediatrics, Karolinska University Hospital, and Karolinska Institute, Stockholm, Sweden; e Women and Children’s Health, Karolinska University Hospital, and Karolinska Institute, Stockholm, Sweden; f Department of Clinical Sciences, Pediatric Surgery, Lund University and Skåne University Hospital, Lund, Sweden; g Department of Clinical Sciences, Pediatric Anesthesiology, Lund University and Skåne University Hospital, Lund, Sweden; h Department of Clinical Sciences, Pediatric Cardiac Surgery, Lund University and Skåne University Hospital, Lund, Sweden

**Keywords:** Tuberous sclerosis, Aortic aneurysms, mTOR signaling, Smooth muscle cell phenotype

## Abstract

Aortic aneurysms are rare manifestations in children with tuberous sclerosis complex (TSC) with life threating implications. Although an association between TSC, aortic and other aneurysms has been recognized, mechanistic insights explaining the pathophysiology behind aneurysm development and genetic aberrations in TSC have so far been lacking. Here, we summarize existing knowledge on aneurysms in TSC and present a case of a 2-year-old boy with an infrarenal aortic aneurysm, successfully treated with open aortic reconstruction. Histologic examination of the excised aneurysm wall showed distortion of vessel wall structure with loss of elastin and a pathologic accumulation of smooth muscle cells. Until now, these pathologic features have puzzled researchers as proliferating smooth muscle cells would rather be expected to preserve vessel wall integrity. Recent reports exploring the biological consequences of the dysregulated intracellular signaling pathways in patients with TSC provide plausible explanations to this paradox, which may support the development of future therapeutic strategies.

Tuberous sclerosis, or tuberous sclerosis complex (TSC), was first recognized as disease entity in the nineteenth century and is one of the most common genetic disorders, now estimated to occur in 1 of 6000 to 10 000 live births.^1^ TSC is characterized by widespread hamartomas commonly found in the brain, heart, skin, eyes, kidney, lung, and liver; skin lesions are most common together with cerebral manifestations followed by renal and retinal involvement.^2^ Although the disease may affect all organs, vascular manifestations are rare. However, both aneurysms and occlusive lesions have been reported in the abdominal and thoracic aorta, in the axillary artery, in the extracranial as well as the intracranial carotid artery, and in the renal arteries.^3^^,^^4^

The pathophysiology behind the disease was unraveled in the 1990s when mutations in the two genes (*TSC1* and *TSC2*) encoding the intracellular proteins hamartin and tuberin were discovered. Hamartin and tuberin normally act as endogenous repressors of mechanistic target of rapamycin (mTOR; previously known as mammalian target of rapamycin) activity, which is a central, anabolic, signaling pathway for cell proliferation, nutrient uptake, transcriptional and translational control. TSC is an autosomal-dominant disorder but with significant contribution by sporadic mutations, which contribute to loss of heterozygosity, inactivation of both alleles for either *TSC1* or *TSC2*, penetrance, and the wide variety of disease manifestations.^2^^,^^5^^,^^6^ These discoveries soon led to the introduction of pharmacotherapy to treat TSC lesions using mTOR inhibitors (rapalogs or rapamycin analogues), to partly substitute for the defective endogenous inhibition. Treatments that have now been established for subependymal giant cell astrocytomas, renal angiomyolipomas, lymphangioleiomyomatosis, and severe epilepsy.^7^ In contrast, there is still no pharmacotherapy for vascular manifestations in TSC, although rapalogs are far from unknown within the cardiovascular field. Since the introduction of the drug-eluting technology, rapalogs have been commonly used in stents and balloons to prevent SMC proliferation in restenosis and intimal hyperplasia after endovascular intervention.^8^ However, recent insights into the cellular and molecular consequences of mTOR hyperactivity in the vessel wall may support the use of rapalogs also for treatment of the vascular manifestations rarely encountered in patients with TSC.

## Case report

**Fig 1:**
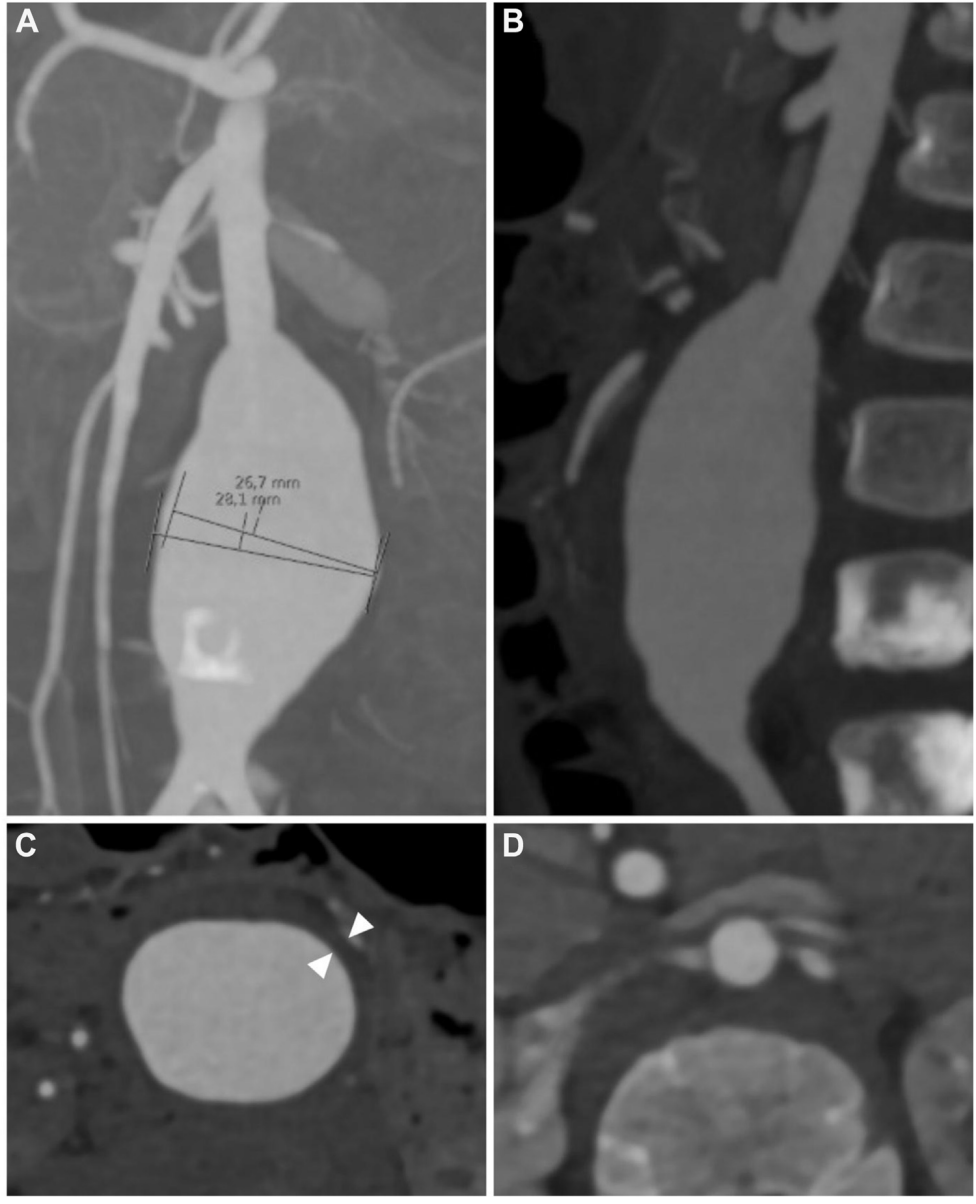
Computed tomography angiography demonstrating a 28-mm-wide infrarenal abdominal aortic aneurysm in a 2-year-old boy. Frontal view **(A)**, sagittal **(B)**, and axial views of the aneurysm **(C)** and the neck **(D)**. Note the massive thickening of the aneurysm wall (**C**; *arrowheads*) in comparison with the normal infrarenal aorta **(D)**.

**Fig 2:**
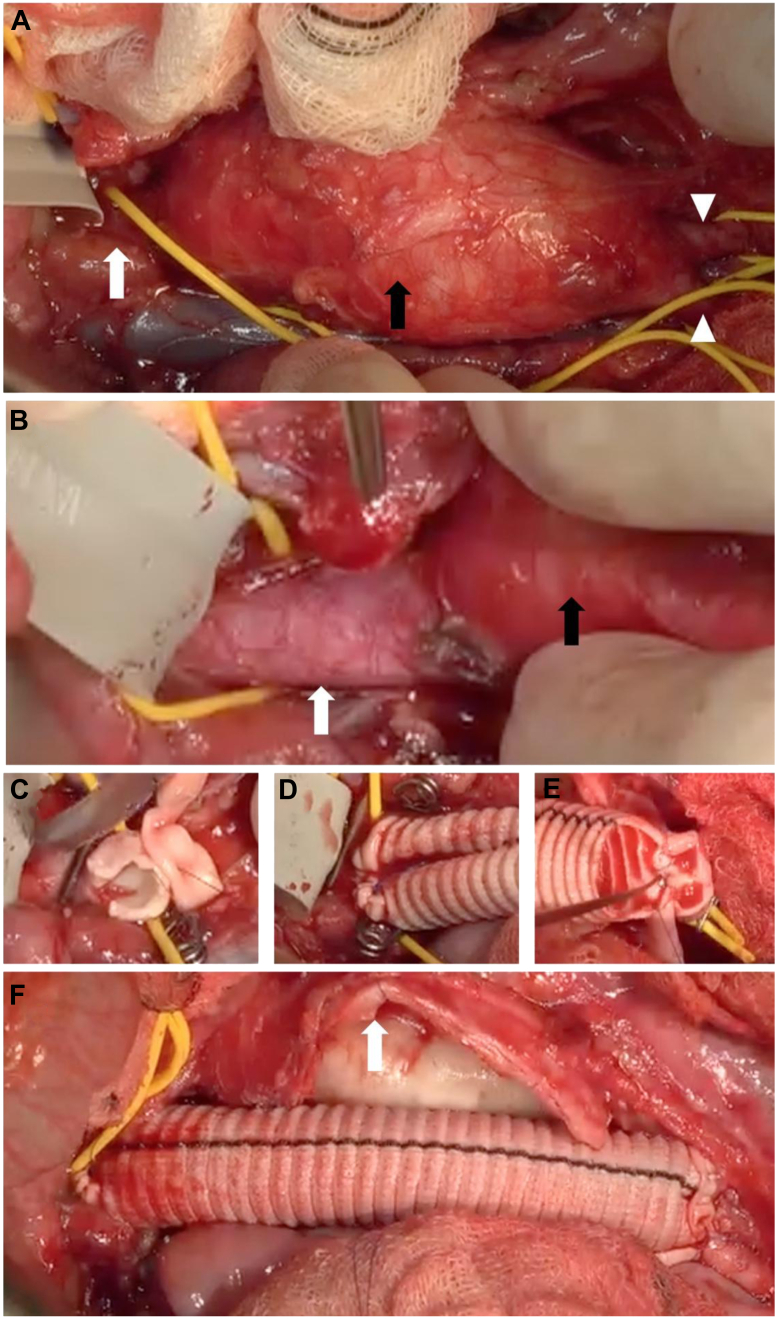
Intraoperative images from aneurysm repair with the aneurysm exposed (**A**; *black arrow*, aneurysm; *white arrow*, infrarenal aortic neck; common iliac arteries, *arrowheads*) and close-up view **(B)** of the pathologically thickened, reddish aneurysm (*black arrow*) compared with the normal aortic neck (*white arrow*). The neck was obliquely transected for a bovine patch-supported proximal anastomosis **(C)** that, when completed, shows the discrepancy between the native aorta and the oversized Dacron graft **(D)**, also seen at the distal anastomosis to the aortic bifurcation **(E)**. Completed straight graft reconstruction with the sectioned, pathologically thickened aneurysm wall exposed (**F**; *white arrow*).

A 2-year-old boy with TSC type 2 accompanied by neurologic (epilepsy), renal (angiomyolipomas), and cardiac (rhabdomyomas) manifestations was incidentally diagnosed with an infrarenal aortic aneurysm during a routine magnetic resonance imaging follow-up of his kidney lesions, which was followed by computed tomography angiography. The aneurysm presented with a 1.3-cm long and 0.6-cm wide infrarenal neck and a fusiform dilatation measuring a maximum diameter of 2.8 cm, ending proximal to the aortic bifurcation. The aneurysm did not contain any mural thrombus and was distinguished by an exceptionally thick vessel wall compared with the normal aorta (Fig 1). Given this rare condition, the available literature was reviewed and aortic repair planned in a multidisciplinary setting with participation of vascular surgery, pediatric cardiothoracic surgery, pediatric surgery, and pediatric anesthesiology. A conventional open transperitoneal approach was chosen, the aneurysm and infrarenal neck exposed, clamps applied to the neck and both common iliac arteries, and the aneurysm opened. The thickened aneurysm wall observed in the preoperative computed tomography scan was verified and proximal and distal transition zones of normal appearing aortic wall cut obliquely for anastomosis. A straight, oversized, 12-mm, preclotted Dacron graft was inserted using running, 6-0 Prolene sutures with a bovine patch support for the anastomosis. Oversizing was chosen to allow continued growth of the native aorta and decrease the requirement for future revision and graft replacement (Fig 2). Patient recovery was uneventful with discharge after 5 days. At the 6- and 9-month follow-up visits, including magnetic resonance imaging of the thoracic and abdominal aorta and intracranial vessels, there was patent reconstruction without signs of recurrent aortic dilatation or other, large vessel or intracranial aneurysms. The patient is currently monitored for the development of new aneurysms and bilateral, thickened renal arteries with discrete luminal narrowing and modest renal hypertension, which is treated pharmacologically.

**Fig 3:**
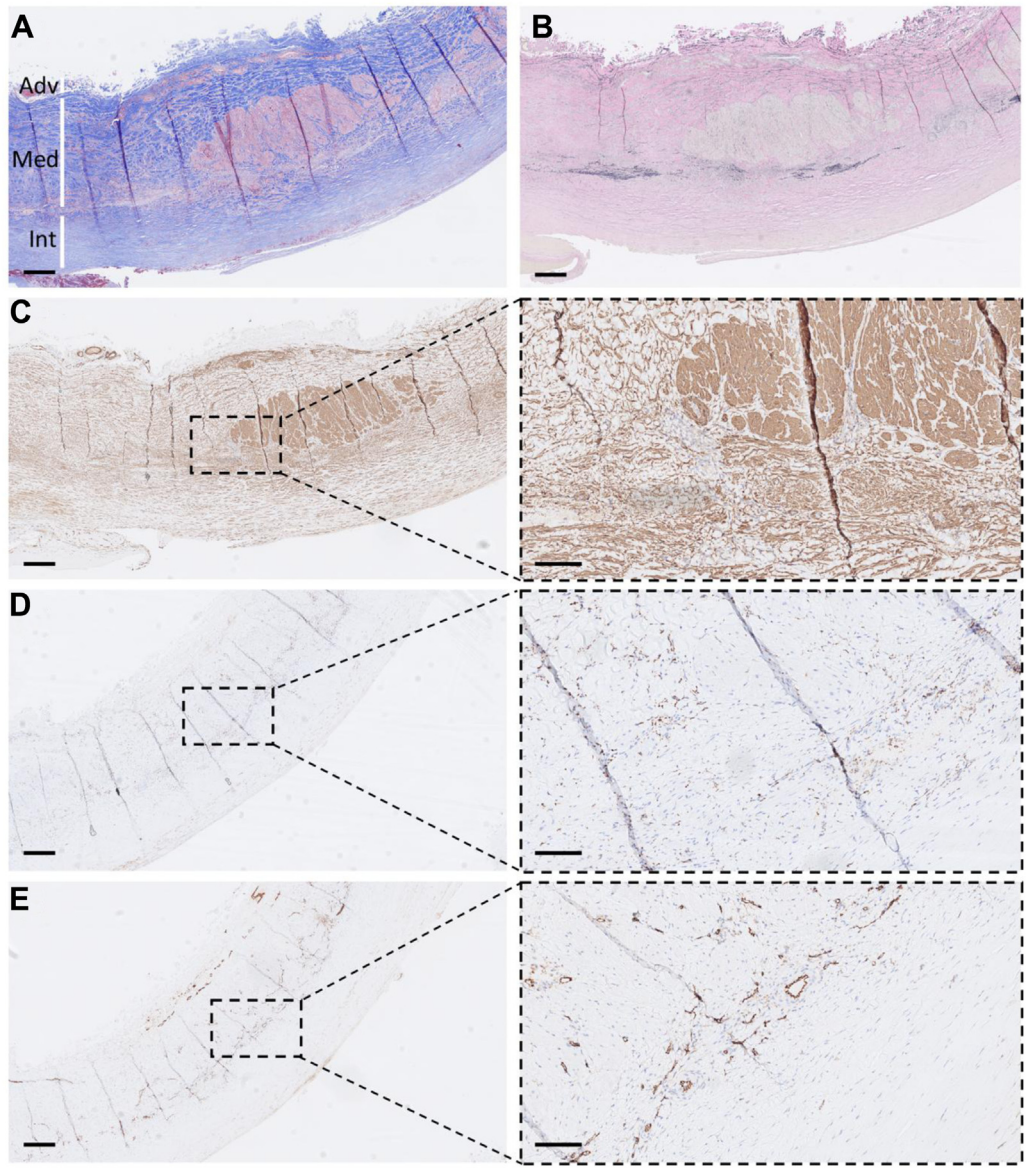
Histologic **(A, B)** and immunohistochemical (IHC; **C–E**) images of aneurysm wall specimens from the presented case. Masson’s Trichrome staining **(A)** demonstrating a thickened aneurysm wall with a distorted, collagen-rich (blue) and a hyperplastic media (*Med*) and intima (*Int*). Elastica van Gieson staining of a parallel section shows dramatically decreased elastin content and complete loss of elastin lamellae **(B)**. IHC for smooth muscle α-actin (SMA) demonstrating dispersed, irregular SMA-positive cells throughout the media and intima with pathologic bundles in the central media (**C**; bar = 0.50 mm; insert, bar = 0.05 mm). IHC for CD68 showing abundant positive cells in the media (**D**; **C**; bar 0.50 = mm; insert bar = 0.05 mm) together with CD31-positive pathologic vascular structures (**E**; **C**; bar = 0.50 mm; insert bar = 0.05 mm). *Adv,* Adventitia.

Histologic examination with conventional and immunohistochemical (IHC) stains was performed of aneurysm wall specimens retained at surgery. Formalin-fixed paraffin-embedded sections stained with Masson’s trichrome showed a collagen-rich vessel wall with a distorted media and a markedly thickened intima. IHC with smooth muscle α-actin staining demonstrated loss of concentric smooth muscle cell (SMC) layers and instead with isolated bundles of smooth muscle actin-positive cells in the media and abundant SMCs dispersed in the intima. Staining with elastica van Gieson showed near complete loss of elastic lamellae in the media and no distinct internal or external elastic lamina separating the vessel wall layers was observed. IHC showed accumulation of inflammatory cells with scattered CD68-positive cells in the media where abundant pathologic vascular structures were observed as estimated by CD31 staining (Fig 3).

## Aortic aneurysms in TSC

Altogether, there are fewer than 40 cases of children with TSC and aortic aneurysms reported in the literature, most commonly described in the infrarenal aorta, and some presenting with rupture and a fatal outcome.^9^, ^10^, ^11^, ^12^, ^13^ Guided by these experiences, it is recommended to search for, and exclude, aortic and large artery aneurysms in children diagnosed with TSC and, when present, to manage with close surveillance or surgery.^9^ In the majority of the reports, open repair has been applied using either Dacron, polytetrafluoroethylene, or biological conduits; an endovascular procedure for a saccular aneurysm in the descending thoracic aorta of a young adult has also been described.^14^ Because recurrent, anastomotic aneurysms and new ones have been encountered in children with TSC, continued surveillance after repair is recommended.^10^

## The biological paradox of aneurysm formation in TSC

In the presented case as well as in previous reports,^9^ a massive increase in aortic wall thickness was observed both in the preoperative computed tomography scan, intraoperatively, and upon histologic examination of aneurysm wall specimens, likely from proliferation and accumulation of SMCs (Fig 1, Fig 2, Fig 3). Traditionally, the activation of SMCs from a differentiated, quiescent, muscle-like cell into a migratory and proliferative fibroblast-like one has been described as a modulation from a contractile into a synthetic or secretory phenotype, as these properties are also accompanied by increased secretion of extracellular matrix components, such as collagen and elastin. This transition normally takes place when SMCs are cultured in vitro as well as in vessel wall repair in numerous vascular pathologies, such as intimal hyperplasia in restenosis and vein graft disease, fibrous cap formation in atherosclerosis, and in transplant vasculopathy.^15^^,^^16^ In contrast, aneurysm formation has rather been associated with extracellular matrix degradation, the loss of SMCs, and vessel wall disintegration.^17^ Thus, the anticipated enhanced proliferative and secretory activity of SMCs in the formation of arterial lesions in TSC as a consequence of hyperactive mTOR signaling seem paradoxical to the observed near complete disappearance of elastin fibers observed in our patient and as described elsewhere by others.^9^^,^^12^^,^^13^ Although less reported, the hyperplastic features of vascular TSC have also be observed as stenotic and occlusive lesions in renal arteries.^4^

Until now, the mechanisms behind these distinct, but contradictory, features of aneurysm formation in TSC have not been understood completely. However, recent experimental work seems to resolve at least some of the confusion.^18^ In this work, the authors showed that tamoxifen-inducible, SMC-specific deletion of *Tsc1* in mice, generating chronic mTOR hyperactivity in SMCs, led to the development of aneurysms in the ascending aorta characterized by typical TSC pathologic features such as aortic wall thickening, SMC proliferation, and loss of elastin fibers, a vascular phenotype that could be rescued by treating the mice with rapamycin. The authors further demonstrated that SMCs in this model displayed distinct phenotypic features characterized by an increased proliferative capacity, yet with defective elastin synthesis, a loss of contractile features, an increased secretion of matrix metalloproteinases, and an increased lysosomal content with an enhanced capacity for macromolecular clearance, as supported by previous studies in heterozygous *Tsc2*-deficient mice.^19^ Even if the association between genotype and vasculopathy is unknown,^20^ these findings in genetically modified mice as well as case reports of patients with either *TSC1* or *TSC2* mutations suggests that aneurysm formation in TSC does not depend on the genotype.^19^^,^^21^^,^^22^

The authors also found indications that the phenotypic switch triggered by enhanced mTOR activity and lysosomal biogenesis was accompanied by a transdifferentiation toward a degradative, macrophage-like phenotype as determined by the acquired expression of a subset of lysosomal macrophage markers (GAL3/Mac2; LAMP2/Mac-3). Previously, SMC transdifferentiation into several cell lineages such as a migratory, proliferative, and secretory phenotype, macrophage-like cells, and osteochondrogenic lineage have been described in atherosclerosis and other arteriopathies; it is conceivable that these findings represent yet another fate of SMCs in arterial disease.^23^ It is possible that this phenotypic switch also occurs in common aneurysm disease; the authors also observed the presence of degradative SMCs of in adult, human, non-TSC, thoracic aneurysms.^18^ Interestingly, we also observed abundant CD68-positive cells in the diseased aortic wall of our patient, representing the accumulation of inflammatory macrophages; however, the contribution of transdifferentiated SMCs similar to those described elsewhere in this article cannot be excluded.

Although a rather convincing mechanism, other cellular and molecular pathways may also operate in TSC arteriopathy, such as pathways activated secondary to the loss of elastin fibers. Dysregulated or defective elastin biosynthesis in both human genetic disorders (eg, Williams syndrome) and in elastin-deficient mice is also accompanied by aneurysms and occlusive lesions, similar to patients with TSC.^24^

## Conclusions and translational perspective

**Fig 4:**
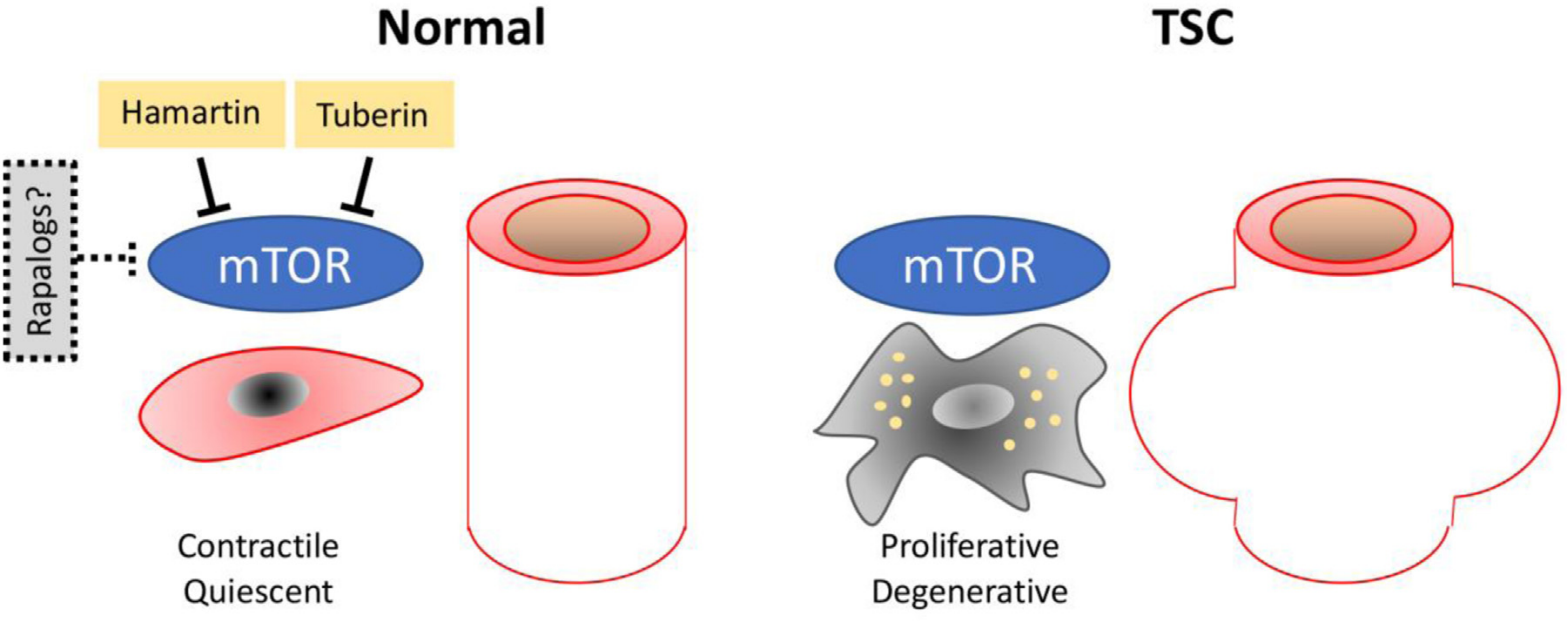
Schematic illustration of possible mechanisms behind aneurysm formation in tuberous sclerosis complex (*TSC*). Under normal conditions, hamartin and tuberin inhibit mechanistic target of rapamycin (*mTOR*) activity, whereas smooth muscle cells (SMCs) reside in a contractile, quiescent state. In TSC, the loss of endogenous inhibition leads to mTOR hyperactivity and a SMC phenotype, which proliferate and increase vessel wall thickness but also degrade the vessel wall extracellular matrix and facilitates aneurysm development. Rapamycin analogues (*rapalogs*) may partly substitute for the loss of endogenous mTOR inhibition and prevent disease development.

From this case report of an abdominal aortic aneurysm in a 2-year-old child with TSC, together with the evolving mechanistic insights into the arteriopathy of the disease, it seems reasonable to speculate how these findings may support translation into clinical practice. Pharmacotherapy with rapalogs for several TSC manifestations are already available, but so far have not been evaluated for the treatment of vascular pathologies, which would be desired in the light of the life-threatening aspects of aortic and intracranial aneurysms. Available drug-eluting stents and balloons with rapamycin analogues seem logical to implement for the treatment of occlusive lesions in TSC and systemic rapalogs could be evaluated for prevention of aneurysm development to avoid or delay surgery in small children, as recently suggested (Fig 4).^21^ In addition, the indications that the mTOR pathway may also operate in adult aneurysm disease deserves further exploration to evaluate whether this pathway may represent a target for future preventive pharmacotherapy of aneurysm growth and rupture as previously reported for experimental aneurysms in rodents.^25^, ^26^, ^27^

## Author contributions

Conception and design: UH

Analysis and interpretation: UH, HB, MD, TB, VPdS, PT

Data collection: UH, HB, PT

Writing the article: UH

Critical revision of the article: UH, HB, MD, TB, VPdS, PT

Final approval of the article: UH, HB, MD, TB, VPdS, PT

Statistical analysis: Not applicable

Obtained funding: UH, PT

Overall responsibility: UH
